# Brucella Endocarditis of the Native Mitral Valve Treated With Antibiotics

**DOI:** 10.7759/cureus.8167

**Published:** 2020-05-17

**Authors:** Muhammad Ali Raza, Komal Ejaz, Daniel Kazmierski

**Affiliations:** 1 Internal Medicine, Conemaugh Memorial Medical Center, Johnstown, USA; 2 Intrenal Medicine, The Wright Center for Graduate Medical Education, Scranton, USA; 3 Internal Medicine, The Wright Center for Graduate Medical Education, Scranton, USA

**Keywords:** brucellosis, brucella endocarditis, native valve endocarditis, mitral valve endocarditis, brucella endocarditis treated with antibiotics alone, brucella endocarditis in pennsylvania

## Abstract

Brucellosis is a rare zoonotic infection with a low annual incidence in the United States. Infective endocarditis secondary to brucellosis involving native or prosthetic valves is contemplated to be an extremely rare entity. As *Brucella* can present with non-specific sign and symptoms, clinicians need to have a higher degree of suspicion of *Brucella* endocarditis in culture-negative endocarditis patients, particularly those who have a history of exposure to farm animals. Timely diagnosis with appropriate management using antibiotics can prevent valvular damage and restore the valve's structural integrity. In this case report, we present a case of culture-negative, serology-proven *Brucella *endocarditis of native mitral valve, with an initial presentation of stroke that was successfully treated with combination antibiotic therapy.

## Introduction

Brucellosis is a worldwide zoonotic infection caused by a gram-negative intracellular bacillus of the genus *Brucella*. Human brucellosisis mainly transmitted via cheese and unpasteurized animal milk. It frequently presents with non-specific symptoms such as fever and malaise, but can also lead to multiorgan failure. Its potential to be employed as a bioterrorism tool coupled with its recent re-emergence has fueled scientific interest and instigated further research about this otherwise uncommonly encountered organism [1]. A multicenter Greek study conducted over 20 years reported the incidence of *Brucella *endocarditis of up to 4% [2]. Although it is rare to have endocarditis caused by *Brucella*, nearly 80% of mortality in brucellosis is secondary to endocarditis [3]. 

## Case presentation

**Figure 1:**
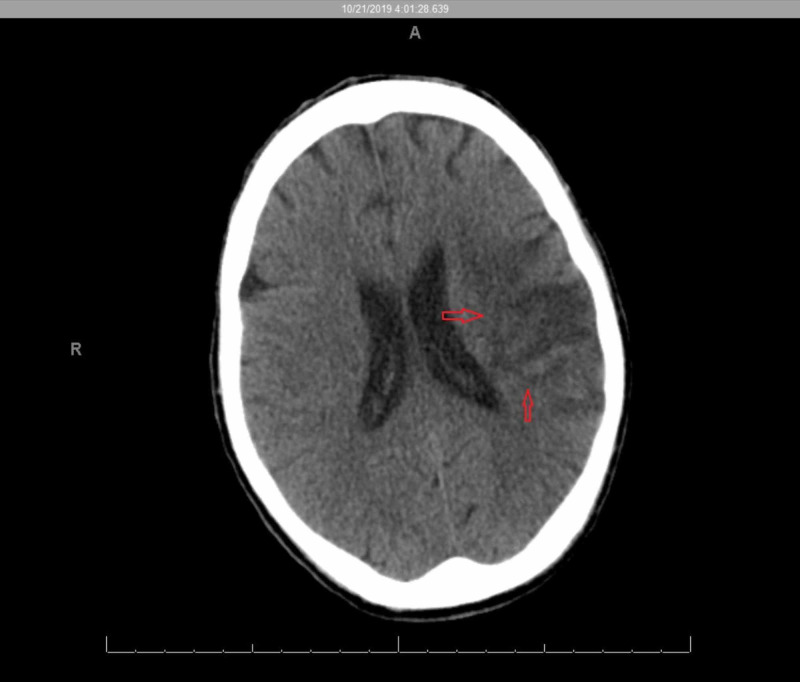
CT of the head without contrast showing middle cerebral artery infarct (red arrows)

A 50-year-old male with a past medical history significant for hepatitis C and intravenous drug abuse with recent incarceration presented to the emergency department (ED) with right-sided weakness and aphasia. As per the family, the patient seemed to be confused and ataxic for about 24 hours before the presentation, and they brought him to the ED after he stopped responding to them. As per the family, the patient resides in a farm in Northeastern Pennsylvania where he breeds sheep. The family denied the patient having any significant cardiac history, diabetes, hypertension or any other known chronic diseases. Pertinent denials included chest pain, shortness of breath, fevers, chills, headache, vision changes, arthralgia, urinary and/or bowel complains. The patient’s surgical and family histories were non-contributory, and he took no medications at home. On presentation, the patient’s Glasgow Coma Scale (GCS) was 8; the patient was confused and aphasic. General physical examination revealed moderate built, malnourished middle-aged male with pallor. Neurological exam was significant for expressive and receptive aphasia, 5/5 muscle strength in left upper and lower extremities and 2/5 in right upper and lower extremities, and profound neglect of the right side of the body. The patient appeared to be agitated when stimulated and did not follow commands. The patient had a normal blood pressure of 112/68 mmHg with a heart rate of 67 beats per minute. A high-pitched systolic murmur was appreciated in the mitral area on auscultation. Other systemic examination did not reveal any abnormality. Routine hematological workup was within normal limits except for mild anemia with Hb of 11.4 g/dl and normal total leukocyte count of 7,000 cells/μl. In the ED, the patient underwent a CT of the head which showed a large middle cerebral artery (MCA) ischemic stroke (Figure 1).

He was deemed to be a poor candidate for thrombectomy and out of tissue plasminogen activator (tPA) window, and hence was admitted to the medical floor for further management. During stroke workup, the patient underwent a transesophageal echocardiography (TEE), which revealed that his mitral valve (MV) was myxomatous and redundant, with mild prolapse and a large mass with associated hypermobile elements attached under the anterior leaflet apparatus and extending to the insertion of the anterior leaflet. Other noteworthy findings included mild central mitral regurgitation (MR) and a severe eccentric jet of MR, which may be due to perforation of the MV leaflet and mild tricuspid regurgitation. The initial workup of infective endocarditis (IE) including methicillin-resistant* Staphylococcus aureus *(MRSA), blood cultures and HIV test was negative, and the patient was treated empirically for culture-negative IE with vancomycin and cefepime. The patient spiked fevers during his hospital stay; however, three repeat blood cultures were negative. Infectious Disease department was consulted and recommended testing for *Bartonella*, Q fever and *Brucella*. *Bartonella* and Q fever serologies were negative, while *Brucella* IgM was elevated at 2.38, with a high *Brucella* antibody agglutination titer of 1:160. Antibiotics were changed to doxycycline 100 mg twice daily for 12 weeks, rifampin 300 mg thrice daily for 12 weeks and intravenous gentamycin 540 mg daily for four weeks. The patient was discharged home with an initial plan for delayed MV replacement. On four-week follow-up, a two-dimensional transthoracic echocardiogram showed resolution of the mass on the anterior leaflet of the MV with no visible vegetation and residual moderate MR. The patient reported improvement in his weakness and was able to walk with a cane and speak a few words. The patient’s clinical improvement was deemed satisfactory, and he was asked to follow up in two months upon completion of his antibiotics course.

## Discussion

Although brucellosis is endemic in developing regions such as the Mediterranean, Middle East, South Asia and South America, it is rare in the developed world with an annual worldwide incidence of approximately 500,000 cases as compared to barely 100 to 200 cases of human brucellosis reported annually in the United States (US) [1,4]. Brucellosis is mostly transmitted to humans via consumption of unpasteurized and infected animal products or inhalation of aerosolized infected particles and through contact with skin or mucous membranes of infected animal tissue [5]. Our patient was a sheep breeder and resided in a farm in Northeastern Pennsylvania, pointing towards a likely source for infection. Human brucellosis is mainly caused by one of the three species: *Brucella melitensis, Brucella abortus* or *Brucella suis* [6]. 

The most common presenting complaint of brucellosis is a fever of unknown origin associated with night sweats, malaise, asthenia and arthralgia. About one-third of patients with brucellosis develop focal complications with osteoarticular complications accounting for more than half [7,8]. Cardiac involvement is rare in brucellosis, of which the most frequently encountered presentation is IE manifested only in about 0.3% to 2% of the patient population [9,10]. 

Despite being rare, brucellosis-associated IE is the most common cause of mortality in patients with brucellosis [10]. In this case, the patient initially presented with stroke and had a new cardiac murmur in the absence of underlying cardiovascular disease, which prompted further workup to rule out underlying cardiac etiologies of stroke, including IE. 

*Brucella* endocarditis involves the aortic valve in 75% of the cases followed by an equal 8.3% involvement of each of the following: MV alone, aortic along with MV and prosthetic valve [11]. Although *Brucella* endocarditis preferentially involves the native aortic valve, the MV is affected if there is pre-existing valvular damage [12]. *Brucella *endocarditis is complicated by the development of myocardial abscesses in 43% of the patient population, which is more common than seen with any other causative organism of endocarditis. Other associated complications include heart failure, embolic phenomenon and disseminated intravascular coagulation [13]. Similarly, the patient in our report presented with an embolic complication in the form of ischemic MCA stroke from underlying *Brucella *endocarditis. 

The diagnosis of *Brucella* IE cannot be made on clinical grounds because of variable clinical presentations. It is mandatory to perform serological and bacteriological testing as definitive diagnosis requires isolating *Brucella* from blood, bone marrow, body fluids or other affected tissue [14]. The gold standard test for detection of *Brucella* is blood culture, which is also one of the major criteria in modified Duke's criteria for IE; however, it has low sensitivity (15%-70%) [1]. Owing to time restraints, the low sensitivity of blood culture, fastidious nature and intracellular location of the organism, the diagnosis of *Brucella* poses a diagnostic challenge thus most physicians rely on indirect serological evidence of brucellosis. Wright agglutination titers of ≥ 1:160 in endemic and ≥ 1:80 in non-endemic regions, such as the US, are very sensitive and specific for active brucellosis in patients with compatible clinical presentation and history of exposure [2,14-16]. In our case, three blood cultures were negative and brucellosis was diagnosed on the basis of serological evidence. Even though the role of serology in the follow-up of human brucellosis is unclear, lower titers correlate with successful treatment [14]. Additionally, echocardiography may also be required to establish the diagnosis and obtain evidence of IE, such as valvular destruction, myocardial or annular abscesses and aneurysm formation [7]. 

The association of *Brucella* IE with valvular abscesses, the formation of large-sized vegetations with a propensity to embolize and the need for several antibiotics required for treatment have mandated surgery to be recognized as the mainstay for its treatment as opposed to IE caused by other organisms [7]. Keshtkar-Jahromi et al. conducted an extensive review of *Brucella* IE cases between 1966 and 2011 and concluded that patients who did not undergo surgery have an increased risk of mortality [17]. However, only a few studies report successful treatment of native or prosthetic valve IE with antibiotics alone [10,18,19]. In these cases, aminoglycosides and doxycycline in combination with rifampicin were used. The recommended antibiotic regimen is a combination of an aminoglycoside (streptomycin or gentamicin) for the first month, in addition to doxycycline and rifampin both for at least 12 weeks [20]. However, if surgery is warranted, then a six-week course of triple antibiotics can be sufficient preoperatively. Moreover, patients should be treated for at least one to three months postoperatively and after titers decrease below 1:160 [15]. In our case, although MV repair was planned initially, the patient had a near-complete resolution of valvular pathology with four weeks of antibiotics course alone. 

## Conclusions

Brucellosis is an uncommon zoonotic infection that should be considered in the differential diagnosis of patients presenting with stroke and fever on unknown origin, especially with a history of exposure to farm animals. *Brucella* endocarditis is a rare complication with high mortality that should be avidly recognized and treated in a timely manner to mitigate complications, such as embolization of cardiac vegetations. Treatment often requires a multidisciplinary approach, including long-term combination antibiotic regimen coupled with surgical intervention.

## References

[1] Pappas G, Papadimitriou P, Akritidis N, Christou L, Tsianos EV (2006). The new global map of human brucellosis. Lancet Infect Dis.

[2] Hadjinikolaou L, Triposkiadis F, Zairis M, Chlapoutakis E, Spyrou P (2001). Successful management of Brucella mellitensis endocarditis with combined medical and surgical approach. Eur J Cardiothorac Surg.

[3] Peery TM, Belter LF (1960). Brucellosis and heart disease: II. Fatal brucellosis: a review of the literature and report of new cases. Am J Pathol.

[4] Bosilkovski M, Dimzova M, Grozdanovski K (2009). Natural history of brucellosis in an endemic region in different time periods. Acta Clin Croat.

[5] Pappas G, Akritidis N, Bosilkovski M, Tsianos E (2005). Medical progress brucellosis. N Engl J Med.

[6] Wise RI (1980). Brucellosis in the United States: past, present, and future. JAMA.

[7] Dahouk SA, Schneider T, Jansen A (2006). Brucella endocarditis in prosthetic valves. Can J Cardiol.

[8] Franco MP, Mulder M, Gilman RH, Smits HL (2007). Human brucellosis. Lancet Infect Dis.

[9] Ferreira P, Gama P, Correia J (2008). Brucella endocarditis: case report and literature review. Rev Port Cardiol.

[10] Lee SA, Kim KH, Shin HS, Lee HS, Choi HM, Kim HK (2014). Successful medical treatment of prosthetic mitral valve endocarditis caused by Brucella abortus. Korean Circ J.

[11] Reguera J, Alarcon A, Miralles F, Pachon J, Juarez C, Colmenero J (2003). Brucella endocarditis: clinical, diagnostic, and therapeutic approach. Eur J Clin Microbiol Infect Dis.

[12] O'Meara JB, Eykyn S, Jenkins BS, Braimbridge MV, Phillips I (1974). Brucella melitensis endocarditis: successful treatment of an infected prosthetic mitral valve. Thorax.

[13] Uddin M, Sanyal S, Mustafa A (1998). The role of aggressive medical therapy along with early surgical intervention in the cure of Brucella endocarditis. Ann Thorac Cardiovasc Surg.

[14] Al Dahouk S, Nöckler K (2011). Implications of laboratory diagnosis on brucellosis therapy. Expert Rev Anti Infect Ther.

[15] Sasmazel A, Baysal A, Fedakar A (2010). Treatment of Brucella endocarditis: 15 years of clinical and surgical experience. Ann Thorac Surg.

[16] Mert A, Ozaras R, Tabak F (2003). The sensitivity and specificity of Brucella agglutination tests. Diagn Microbiol Infect Dis.

[17] Keshtkar-Jahromi M, Razavi S-M, Gholamin S, Keshtkar-Jahromi M, Hossain M, Sajadi MM (2012). Medical versus medical and surgical treatment for Brucella endocarditis. Ann Thorac Surg.

[18] Mert A, Kocak F, Ozaras R (2002). The role of antibiotic treatment alone for the management of Brucella endocarditis in adults: a case report and literature review. Ann Thorac Cardiovasc Surg.

[19] Murdaca G, Colombo BM, Caiti M, Cagnati P, Massa G, Puppo F (2007). Remission of Brucella endocarditis in a patient with mitral valve mechanical prosthesis by antibiotic therapy alone: a case report. Int J Cardiol.

[20] Koruk ST, Erdem H, Koruk I (2012). Management of Brucella endocarditis: results of the Gulhane study. Int J Antimicrob Agents.

